# Rootstock-induced molecular responses associated with drought tolerance in sweet orange as revealed by RNA-Seq

**DOI:** 10.1186/s12864-019-5481-z

**Published:** 2019-02-06

**Authors:** Luana P. Gonçalves, Raquel L. Boscariol Camargo, Marco Aurélio Takita, Marcos A. Machado, Walter S. dos Soares Filho, Marcio G. C. Costa

**Affiliations:** 1 Centro de Biotecnologia e Genética, Departamento de Ciências Biológicas, Universidade Estadual de Santa Cruz, Ilhéus, BA 45662-900 Brazil; 2 Centro APTA Citros Sylvio Moreira, Instituto Agronômico, Cordeirópolis, SP 13490-970 Brazil; 3 Embrapa Mandioca e Fruticultura, Cruz das Almas, BA 44380-000 Brazil

**Keywords:** Citrus, Transcriptome, Water deficit, Abiotic stress

## Abstract

**Background:**

Citrus plants are commercially propagated by grafting, with the rootstock variety influencing a number of horticultural traits, including drought tolerance. Among the different rootstock varieties available for citrus propagation, ‘Rangpur’ lime is known to confer enhanced tolerance to drought as compared to other citrus rootstocks. The objective of this study was to investigate the poorly understood molecular responses underlying the rootstock-induced drought tolerance in sweet orange.

**Results:**

RNA-Seq transcriptome analysis was carried out in leaves of sweet orange grafted on ‘Rangpur’ lime subjected to control and drought-stress treatments, under greenhouse conditions, using the Illumina HiSeq platform. A total of 41,827 unique transcripts were identified, among which 1764 transcripts showed significant variation (*P* ≤ 0.001) between the treatments, with 1081 genes induced and 683 repressed by drought-stress treatment. The transcripts were distributed in 44 different categories of cellular component, molecular function and biological process. Several genes related to cell metabolism, including those involved in the metabolisms of cell wall, carbohydrates and antioxidants, light reactions, biotic and abiotic stress responses, as well as genes coding for transcription factors (TFs), protein kinases (PKs) and proteins involved in the abscisic acid (ABA) and ethylene signaling pathways, were differentially regulated by drought stress. RNA-Seq data were validated by quantitative real-time PCR (qPCR) analysis and comparative analysis of expression of the selected genes between sweet orange grafted on drought-tolerant and -sensitive rootstocks revealed new candidate genes for drought tolerance in citrus.

**Conclusions:**

In conclusion, our results showed that only a relatively small but functionally diverse fraction of the sweet orange transcriptome, with functions in metabolism, cellular responses and regulation, was differentially regulated by drought stress. The data suggest that the rootstock-induced drought tolerance in sweet orange includes the transcriptional activation of genes related to the cell wall, soluble carbohydrate and antioxidant metabolisms, biotic and abiotic stress responses, TFs, PKs and ABA signaling pathway, and the downregulation of genes involved in the starch metabolism, light reactions and ethylene signaling. Future efforts to elucidate their functional roles and explore their potential in the citrus genetic improvement should benefit from this data.

**Electronic supplementary material:**

The online version of this article (10.1186/s12864-019-5481-z) contains supplementary material, which is available to authorized users.

## Background

Citrus are the most economically important fruit crops in the world, with sweet orange accounting for nearly 60% of the total citrus production [1]. Citrus cultivation is limited to tropical and subtropical regions of the world, where drought is one of the major environmental constraints limiting its production [2]. Such a constraint is expected to increase in intensity, frequency, and geographic extent as a result of global climate change. Therefore, understanding the physiological mechanisms of drought tolerance, as well as the correlated molecular responses and associated genes, is an urgent and demanding issue for the adaptation of citrus crops to the present and future climate.

Studies on plant-water relations showed that plants have evolved two major mechanisms of drought resistance: stress avoidance and tolerance. Stress avoidance refers to the ability of the plants to maintain a suitable water status in their tissues under progressive soil water deficit. Avoidance responses rely on different strategies that restrict further water loss, including stomatal closure, reduced leaf area and growth, deep rooting and increased water use efficiency (WUE) [3]. Conversely, stress tolerance refers to the plant’s ability to partially dehydrate but remain viable and grow again when rainfall resumes. The primary strategies that contribute to drought tolerance include changes in tissue elasticity (e.g. bulk elastic modulus, ε), osmotic adjustment and efficient antioxidant capacity [4].

Various experimental evidences have shown that drought tolerance in *Citrus* spp., related genera (e.g. *Poncirus*) and their hybrids (e.g. citranges and citrumelos) is based mainly on avoidance mechanisms that include stomatal closure, and hence decreased transpiration and CO_2_ assimilation [5–9], reduced leaf area, vegetative growth and yield [10–12]. On the other hand, some evidences suggest that citrus plants also exhibit some tolerance mechanisms to drought, such as osmotic [5, 7] and cell wall elastic [13] adjustments. Another striking feature observed in citrus under field conditions is that drought tolerance is a characteristic usually conferred by the rootstock [14, 15]. For instance, the rootstock ‘Rangpur’ lime is known to confer enhanced drought tolerance to sweet orange scions under field conditions as compared with other citrus rootstocks, such as ‘Swingle’ citrumelo, ‘Sunki’ mandarin, ‘Cleopatra’ mandarin and *Poncirus trifoliata* [16]. For this reason, it remains the most preferred rootstock used in the Brazilian citrus industry, despite of its susceptibility to economically important diseases such as citrus blight and citrus sudden death [17]. The outstanding performance of ‘Rangpur’ has been related to its high root hydraulic conductivity [18], enhanced root growth [19] and remobilization of carbohydrate reserves to the roots [9] under drought-stress conditions.

The response of citrus to drought stress is poorly understood at molecular level. In a first attempt, expressed-sequence-tag (EST) analysis of 4130 valid reads from roots of ‘Rangpur’ seedlings cultivated in hydroponic conditions with and without drought stress using polyethylene glycol-6000 (PEG-6000) resulted in the identification of 40 differentially expressed genes [20]. These included homologues to well known genes involved in drought-stress response, such as aquaporins, chaperones, dehydrin, proteases, sucrose synthase and proline-related synthase, as well as homologues to other previously unrelated genes involved with cell energy [e.g. glyceraldehyde-3-phosphate dehydrogenase (GAP3C), phosphoenol pyruvate carboxykinase (PEPCK), glycogenin glucosyltransferase (GGT)], protein synthesis (e.g. ribosomal proteins) and cellular transport (e.g. nodulin-like protein). In another study, a citrus cDNA microarray of ~ 6000 genes was used to identify, within 5–24 h after transplantation from wet sand to dry sand, 289 drought-induced and 91 drought-repressed genes in roots and 573 drought-induced and 488 drought-repressed genes in leaves of ‘Clemenules’ mandarin (*C. clementina* cv. Clementina) grafted on ‘Cleopatra’ mandarin (*C. reshni* Hort. ex Tan.) [21]. The products of the stress-inducible genes identified also included well known proteins associated with stress response, such as those involved in the lysine catabolism, proline and raffinose synthesis, hydrogen peroxide reduction, vacuolar malate transport, rare-cold-inducible 2 (RCI2) proteolipids and cell protection (e.g. osmotin, dehydrins and heat-shock proteins). Novel drought-inducible genes were also identified in this study, including those encoding miraculin, β-carotene hydroxylase, oleoyl desaturase, small subunit ribosomal protein S13A (RPS13A) and constitutive triple response 1 (CTR1) protein kinase. These previous studies not only reinforced the earlier findings about the commonly represented classes of genes induced by drought stress in different plant species, but also suggested that the molecular responses of citrus plants to drought stress may also include the induction of new genes with functions in cell energy, synthesis of proteins, zeaxanthin and linolenoyls, inhibition of proteases and ethylene signaling.

More recently, we have unveiled some functional leaf traits favoring drought tolerance in sweet orange as induced by citrus rootstocks of contrasting drought response [22]. In contrast to plants grafted onto the drought-sensitive rootstock ‘Flying Dragon’ trifoliate orange (*P. trifoliata* L. Raf.), those grafted onto the drought-tolerant rootstock ‘Rangpur’ exhibited a decreased bulk elastic modulus (ε), low relative water content at turgor loss point (RWC^TLP^) and efficient antioxidant capacity [22]. The molecular basis of these adaptive responses of drought tolerance remains to be elucidated. Our hypothesis is that the drought-tolerant rootstock, but not the drought-sensitive one, induces key components of regulatory networks (e.g. transcription factors, protein kinases and protein phosphatases) controlling the expression of genes involved in cell wall synthesis and modification, osmolyte biosynthesis, antioxidant metabolism, among other processes. This hypothesis is supported by evidences that grafting can determine stock-specific transcript (mRNAs and miRNAs) concentration changes in scions, as recently shown in grapevine [23, 24], cucumber and pumpkin [25].

To advance our understanding on the molecular responses underlying the rootstock-induced drought tolerance in sweet orange, we report here, for the first time, the results of an RNA sequencing (RNA-Seq) transcriptome analysis using Illumina deep sequencing of RNA populations obtained from control and drought-stressed leaves of ‘Rangpur’-grafted sweet orange collected in our previous study [22]. Quantitative Real-Time-PCR (qPCR) validation of the differentially expressed transcripts from RNA-Seq and their comparative abundance with the ‘Flying Dragon’-grafted sweet orange revealed novel candidate genes associated with drought-stress tolerance in citrus.

## Results

### RNA sequencing and differential gene expression analysis

A total of three RNA libraries from leaf samples of control (irrigated; LC1) and drought-stressed (LC3 and LC4) ‘Rangpur’-grafted sweet orange were sequenced as 100 bp paired-end runs on an Illumina HiSeq platform. The libraries produced from 5.98 (LC1) to 6.74 (LC3) Gb raw data from paired-end (PE) reads, with a single read length of 101 bp, a Q20 percentage (percentage of sequences with sequencing error rate lower than 1%) over 96% and an unknown base percentage (N) of 0.005% (Additional file 1: Table S1). These data showed that the throughput and sequencing quality were high enough for further analysis.

The reads were aligned with the *Citrus sinensis* reference genome and the expression levels of a total of 41,827 unique transcripts were quantified based on the Cuffdiff analysis. A total of 1764 transcripts showed significant (*P* ≤ 0.001) variation (≥ + 2.0 or ≤ − 2.0 fold-change) of expression between the control and drought-stress treatments, with 1081 genes induced and 683 repressed by drought stress (Additional file 2: Table S2-1 and Additional file 3: Table S2-2).

**Fig. 1:**
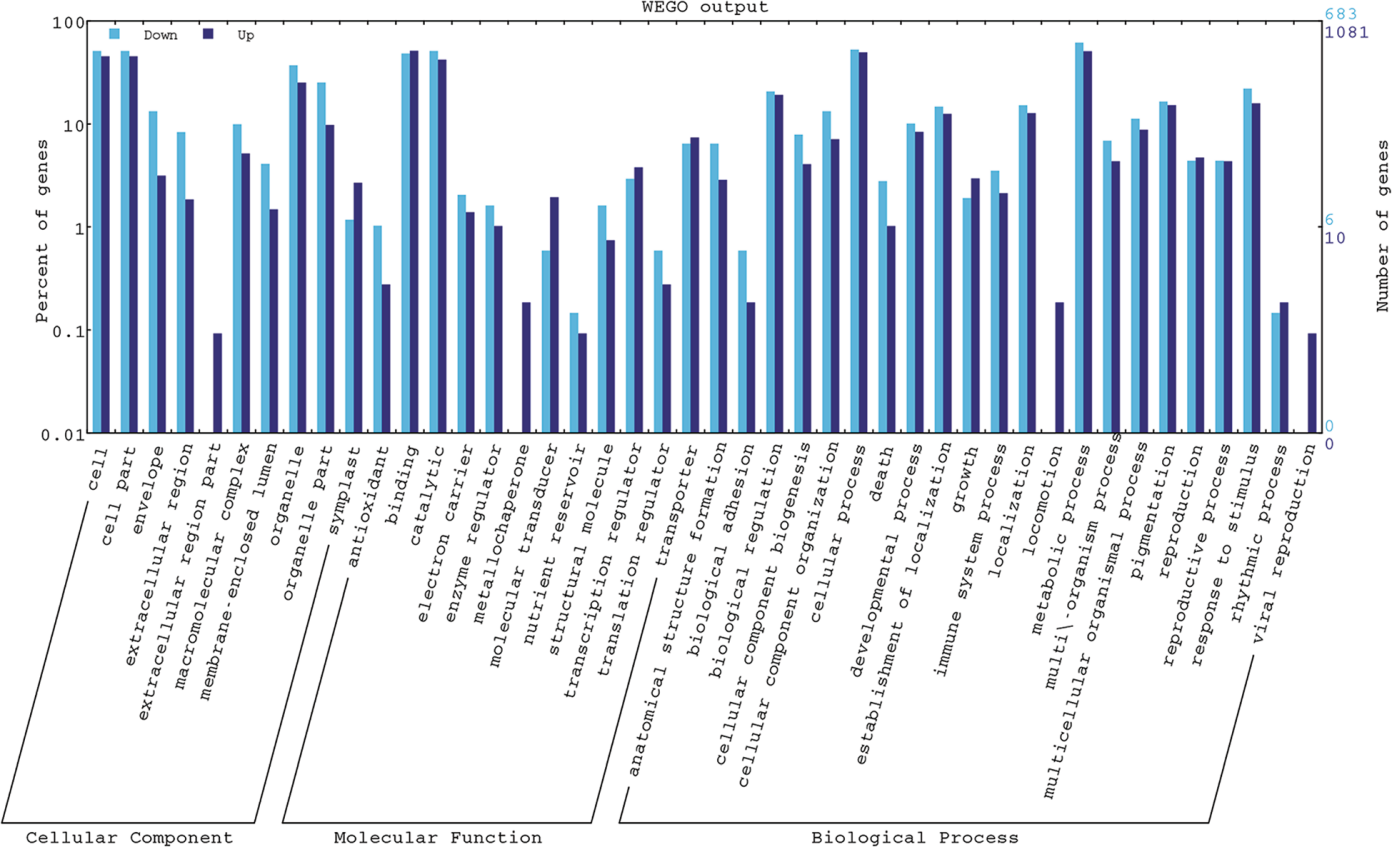
Functional categorization of the differentially expressed genes in leaves of drought-stressed sweet orange (*Citrus sinensis*) grafted on ‘Rangpur’ lime. Categorization was performed using BGI WEGO

The differentially expressed genes were categorized using Gene Ontology (GO). Based on their similarity, the transcripts were distributed into 44 different categories of cellular component, molecular function and biological process (Fig. 1). In terms of cellular components, drought stress primarily affected cell part and organelle part. The molecular functions of differentially expressed genes (DEGs) were most related to binding and catalytic. Among biological processes, cellular process, metabolic process, biological regulation and response to stimulus contained the most DEGs.

**Fig. 2:**
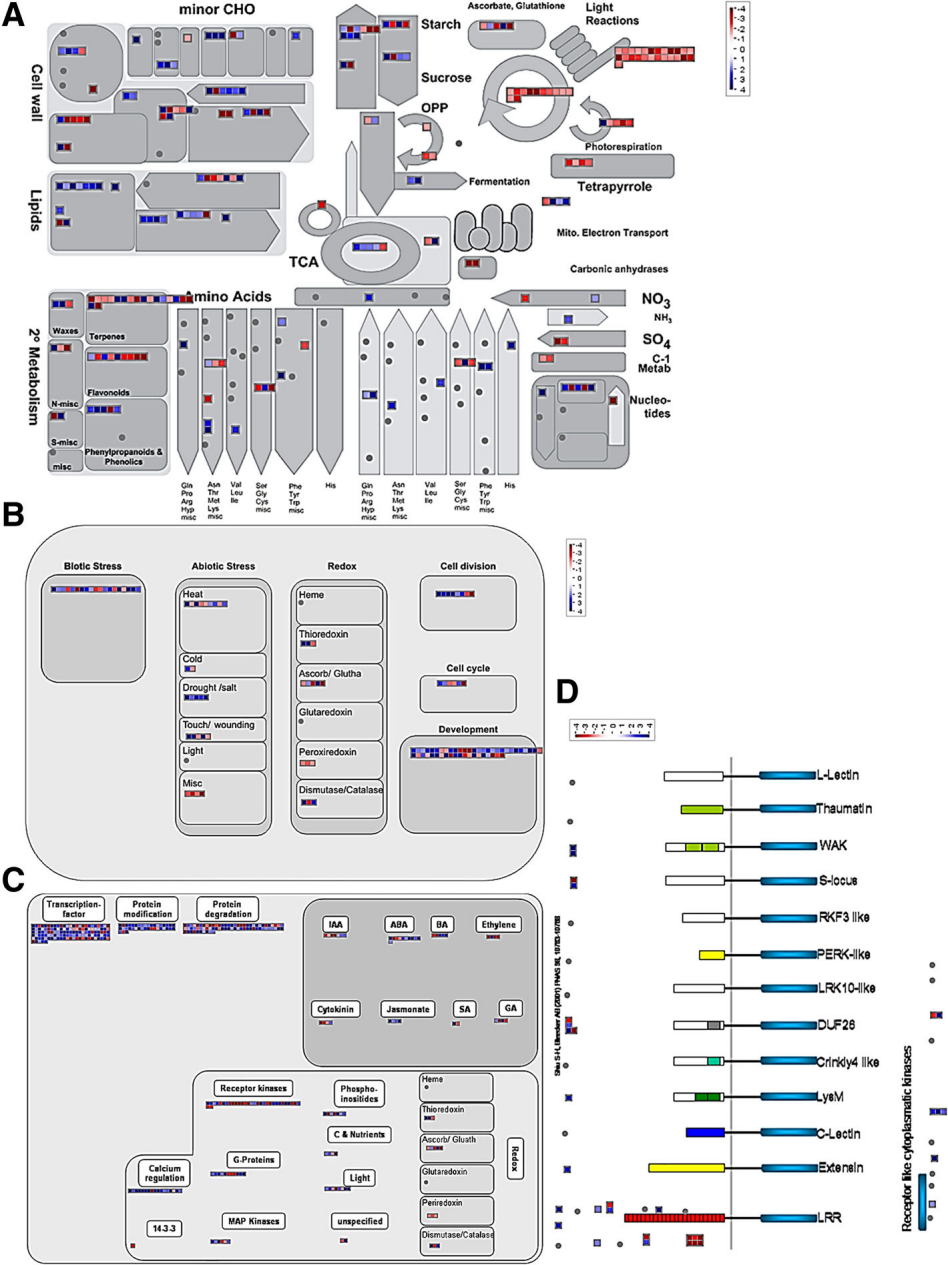
Overview of the differentially expressed genes related to metabolic pathways (**a**), cellular responses (**b**), regulation (**c**) and receptor-like kinases (**d**) in leaves of drought-stressed sweet orange (*Citrus sinensis*) grafted on ‘Rangpur’ lime. Genes that were significantly induced are shown in blue and repressed genes shown in red. Abbreviations/definitions: LRR, leucine-rich repeats; Extensin, RLK with extensin motif; LysM, RLKs with lysine motif; C-lectin, RLKs with lectin-like motifs; Crinkly 4-like, RLKs with crinkly4-like domains; DUF26, domain of unknown function 26; LRK 10-like, RLK gene linked to Lr10 locus; L-lectin, RLKs with lectin-binding domains; PERK-like, proline-rich extensin-like kinase; S-locus, RLK with S-domain similar to S-locus glycoproteins; RKF3-like, receptor-like kinase in flowers 3; Thaumatin, RLK-like thaumatin protein; WAK, wall-associated kinase

Views of the functional groups that were significantly regulated by drought stress were generated using the MapMan 3.5.1R2 software [26]. These included several genes related to metabolism, cellular response, regulation and receptor-like kinases (Fig. 2 and Additional files 4, 5, 6, 7: Tables S3, S4, S5, S6).

The accuracy of the RNA-Seq expression profiles was further validated by qPCR analysis. Fifteen genes encoding cell wall-related proteins, transcription factors (TFs) and protein kinases (PKs) were selected to validate their expression (Table 1). There was a significant (*P* = 0.0048) positive correlation (Pearson coefficient of 0.75) in the expression profiles between RNA-seq and qPCR data, indicating the reliability of the RNA-Seq data.

**Table 1:** Validation of differentially expressed genes related to cell wall metabolism, transcription factors and receptor-like kinases in leaves of drought-stressed sweet orange (*Citrus sinensis*) grafted on ‘Rangpur’ lime

ID citrus	Gene description	RNAseq fold change (*P* ≤ 0.001)	qPCR log_2_ fold change*
Cell wall metabolism			
Orange1.1g025810m	Expansin-Like B1	36.764	3.716
Orange1.1g013402m	Polygalacturonase (pectinase)	14.367	0.539
Orange1.1g030669m	Arabinogalactan protein 1	3.787	1.406
Orange1.1g047288m	Pectinesterase inhibitor 6-like	−4.516	−0.579
Orange1.1g029350m	Proline-Rich Protein 4	−6.134	0.219
Orange1.1g009682m	Pectinesterase inhibitor 54-like	−11.369	0.205
Orange1.1g025025m	Expansin A4	−12.863	−1.552
Orange1.1g007089m	β-Xylosidade 1	−14.818	0.317
Orange1.1g011204m	Pectin lyase-1 like	−77.398	−2.640
Transcription factor			
Orange1.1g024111m	bHLH BEE 3-like	12.023	5.925
Orange1.1g025097m	WRKY transcription factor 40	14.04	3.412
Orange1.1g021910m	NAC transcription factor 2	10.171	2.673
Receptor-like kinase			
Orange1.1 g031436	Wall-associated receptor kinase-like 8	7.145	−2.482
Orange1.1 g026214	Wall-associated receptor kinase-like 2	8.891	1.386
Orange1.1 g041671	Wall-associated receptor kinase 2	4.977	−0.477

*r = 0.75 (*P* = 0.0048)

### DEGs related to the cell wall metabolism

Thirty-two DEGs putatively involved in the cell wall metabolism were identified in the present study, including those encoding for cell wall precursors (e.g. UDP-glucose) and proteins involved in cellulose synthesis (e.g. cellulose synthases - CESAs), cell wall biogenesis (e.g. arabinogalactans and galactosyltransferases) and modification (e.g. expansins, pectinesterases and their inhibitors, polygalacturonase and pectin lyases) (Table 2).

**Table 2:** Differentially expressed genes related to the cell wall metabolism in leaves of drought-stressed sweet orange (*Citrus sinensis*) grafted on ‘Rangpur’ lime

ID *Citrus*	Gene description	Log_2_ fold change
Cell wall precursors		
Orange1.1g007727m	UDP-sugar pyrophosphorylase	5.581
Orange1.1g001947m	ARA1 (ARABINOSE KINASE 1)	3.566
Orange1.1g011650m	UDP-glucose 6	2.938
Orange1.1g040584m	UDP-glucose 4-epimerase 5	2.7
Orange1.1g005949m	UDP-L-rhamnose synthase	2.664
Orange1.1g019795m	UDP-D-glucose 4-epimerase 1	−6.302
Cellulose synthesis		
Orange1.1g001382m	Cellulose synthase 6	10.771
Orange1.1g001421m	Cellulose synthase 3	5.953
Orange1.1g001373m	Cellulose synthase 9	4.004
Orange1.1g026442m	Cellulose synthase family protein	−2.207
Orange1.1g009524m	Cellulose synthase-like protein e1-like	−2.554
Orange1.1g006639m	Cellulose synthase family protein	−3.725
Orange1.1g004692m	Cellulose synthase 8	−4.088
Cell wall biogenesis		
Orange1.1g030669m	AGP1 (Arabinogalactan Protein 1)	3.787
Orange1.1g009630m	Galactosyltransferase family protein	3.03
Orange1.1g025959m	FLA13 (Fasciclin-Like Arabinogalactan Protein 13 Precursor)	2.805
Orange1.1g007197m	Xyloglucan galactosyltransferase katamari1	2.539
Orange1.1g031862m	AGP2 (Arabinogalactan Protein 2)	2.509
Orange1.1g044183m	Fasciclin-like arabinogalactan protein 4-like	−2.618
Orange1.1g029405m	PRP4 (Proline-Rich Protein 4)	−6.134
Cell wall modification		
Orange1.1g025810m	Expansin-like B1	36.764
Orange1.1g013402m	Probable Polygalacturonase-like	14.367
Orange1.1g008722m	Pectin Esterase Inhibitor-like	3.415
Orange1.1g005706m	Rhamnogalacturonate lyase b-like isoform ×2	2.82
Orange1.1g013734m	Protein notum homolog isoform ×2	−3.309
Orange1.1g018173m	Pectinesterase 3-like	−3.599
Orange1.1g011170m	Alpha-l-fucosidase 1-like	−4.527
Orange1.1g009682m	Probable pectinesterase pectinesterase inhibitor 54-like	−11.369
Orange1.1g025025m	Expansin A 4	−12.863
Orange1.1g012308m	Pectin lyase-like superfamily protein isoform 1	−13.862
Orange1.1g007089m	BETA-d-xylosidase family protein	−14.818
Orange1.1g011204m	Pectin lyase-like superfamily protein isoform 1	−77.398

To advance in their validation as candidate genes for drought tolerance in citrus, some of these DEGs were selected for further comparative expression analysis between sweet orange plants grafted on the drought-tolerant ‘Rangpur’ lime and -sensitive ‘Flying Dragon’ trifoliate orange. Most of the cell wall-related DEGs analyzed showed significantly higher expression levels in drought-stressed sweet orange grafted on the drought-tolerant (‘Rangpur’) than on the drought-sensitive (‘Flying Dragon’) rootstock (Table 3). These include DEGs coding for expansin-like B1 (EXLB1), polygalacturonase (PG), arabinogalactan protein 1 (AGP1), pectinesterase inhibitor 6-like (PEI 6-like), proline-rich protein 4 (PRP4), PEI 54-like, β-xylosidase 1 (BXL1) and pectin lyase-1 like (PLL1).

**Table 3:** Quantitative Real-Time-PCR (qPCR) expression analysis of some drought-regulated genes in leaves of drought-stressed sweet orange (*Citrus sinensis*) grafted on ‘Rangpur’ lime and ‘Flying Dragon’ trifoliate orange

ID citrus	Gene description	Log_2_ fold change (drought/control)	
		‘Rangpur’	‘Flying Dragon’
Cell wall metabolism			
Orange1.1g025810m	Expansin-Like B1	3.716	0.871
Orange1.1g013402m	Polygalacturonase (pectinase)	0.539	−0.636
Orange1.1g030669m	Arabinogalactan protein 1	1.406	−0.277
Orange1.1g047288m	Pectinesterase inhibitor 6-like	−0.579	−2.101
Orange1.1g029350m	Proline-Rich Protein 4	0.219	−2.826
Orange1.1g009682m	Pectinesterase inhibitor 54-like	0.205	−2.439
Orange1.1g025025m	Expansin A4	−1.552	0.272
Orange1.1g007089m	β-Xylosidase 1	0.317	−1.833
Orange1.1g011204m	Pectin lyase-1 like	−2.640	−3.699
Transcription factor			
Orange1.1g024111m	bHLH BEE 3-like	5.925	0.600
Orange1.1g025097m	WRKY transcription factor 40	3.412	−0.785
Orange1.1g021910m	NAC transcription factor 2	2.673	0.945
Receptor-like kinase			
Orange1.1 g031436	Wall-associated receptor kinase-like 8	−2.482	0.889
Orange1.1 g026214	Wall-associated receptor kinase-like 2	1.386	−2.195
Orange1.1 g041671	Wall-associated receptor kinase 2	−0.477	−1.276

### DEGs related to other physiological and molecular responses of drought tolerance in citrus

We have found several DEGs related to other physiological and molecular responses of drought tolerance previously reported in citrus. These include DEGs coding for enzymes involved in the metabolisms of carbohydrates [beta-fructosidase 3 (BFRUCT3), sucrose phosphate synthase 1F (SPS1F), sucrose synthase 3 and 4 (SUS3 and SUS4), hexokinase 2 (HXK2), ADP-glucose pyrophosphorylases 1 and 3 (APS1 and APL3), granule bound starch synthase 1 (GBSS1) and beta-amylase 3 (BAM3)], antioxidants [superoxide dismutase (SOD) and ascorbate peroxidase (APX)], carotenoids [chloroplast beta-carotene hydroxylase (CHY1)] and light reactions (PSBA, PSBO, PSBP and CAB) (Fig. 2a and Additional file 4: Table S3). They also include DEGs related to biotic and abiotic stress response [heat shock proteins (HSPs), early responsive to dehydration (ERD), putrescine N-methyltransferases (PMTs) and miraculin] (Fig. 2b and Additional file 5: Table S4) and hormone signaling (HVA22, MARD1, AREB2, PP2Cs, PYR/PYL/RCAR and ERT2) (Fig. 2c and Additional file 6: Table S5).

### DEGs related to transcription factors (TFs)

We have found 120 DEGs coding for TFs, distributed in several large TF families, including MYB, AP2/ERF, WRKY, bZIP, NAC, zinc finger (ZF) and basic helix-loop-helix (bHLH), among others (Fig. 2c; Table 4; Additional file 7: Table S6). ZF and MYB were the TF families with the largest number of DEGs, 23 and 18 genes, respectively (Table 4). Most DEGs in the different TF families were upregulated by drought stress, except for those belonging to the auxin response factor (ARF) and homeobox (HB) families, in which most of the DEGs were downregulated by drought stress (Table 4).

**Table 4:** Number of differentially expressed genes belonging to the different transcription factor and protein kinase gene families in leaves of drought-stressed sweet orange (*Citrus sinensis*) grafted on ‘Rangpur’ lime

Gene families	Downregulated	Upregulated
Transcription factor		
MYB domain transcription factor family	6	12
WRKY domain transcription factor family	1	5
bZIP transcription factor Family	1	7
HSF, Heat shock transcription	2	1
NAC transcription factor Family	0	7
Trihelix transcription factor	1	3
Zinc finger Family	7	16
ARF, Auxin response factor family	5	4
Aux/IAA Family	0	2
bHLH, Basic helix-loop-helix family	2	6
AP2/EREBP, APETALA2/Ethylene-responsive element binding protein family	2	5
HB, Homeobox transcription factor family	7	1
NF-Y, Nuclear Factor Y family	1	2
Protein kinase		
Leucine Rich Repeat	26	20
Protein tyrosine kinase	1	5
Serine/threonine-protein kinase	0	10
Histidine kinase	0	3
PI3K, Phosphoinositide 3-kinase	0	5
MAP kinase	1	5
Aspartate kinase	0	2
PIP5K, Phosphatidylinositol-4-phosphate 5-Kinase	0	2
Cdc2-related protein kinase	3	1
Wall-associated kinase	0	3
REC, Response regulator receiver domain	0	3

Three of the most upregulated TF DEGs found in the present study (*BEE 3-LIKE*, *WRKY40* and *NAC2*) were selected for further expression validation (Table 1) and comparative expression analysis between sweet orange plants grafted on the drought-tolerant (‘Rangpur’) and -sensitive (‘Flying Dragon’) rootstocks (Table 3). qPCR analysis confirmed all of them as highly induced *TF* genes by drought stress in sweet orange grafted on ‘Rangpur’ (Table 1). On the other hand, no significant changes in the level of expression of these *TF*s were observed in drought-stressed sweet orange grafted on ‘Flying Dragon’ (Table 3).

### DEGs related to protein kinases (PKs)

Ninety DEGs coding for PKs distributed in several families were identified in the present study (Fig. 2d; Table 4; Additional file 7: Table S6). The largest number of DEGs was found in the leucine rich repeat (LRR) and Serine/Threonine-protein kinase (STK) families, with 46 and 10 genes, respectively (Table 4). As also observed for TF DEGs, most DEGs coding for PKs were upregulated by drought stress, except for those of LRR and Cdc2-related protein kinase families, whose most DEGs were downregulated by drought stress (Table 4).

Three *PK*s coding for wall associated kinases (WAKs) were chosen in the present study for validation of expression, given their putative involvement in cell wall signaling pathways. qPCR analysis confirmed a significant induction by drought stress for only one of them, *WAKL2*, whereas the other WAKs (*WAKL8* and *WAK2*) were downregulated by drought (Table 1). Comparative expression analysis between sweet orange grafted on the drought-tolerant (‘Rangpur’) and -sensitive (‘Flying Dragon’) rootstocks showed that all *WAK*s analyzed were differentially regulated by drought according to the rootstock (Table 3). *WAKL2* was upregulated in the drought-tolerant, but downregulated in the drought-sensitive rootstock, whereas the opposite was observed for *WAKL8*. Conversely, *WAK2* was downregulated by drought stress in both rootstocks, but at different levels (Table 3).

## Discussion

In this study, drought-responsive genes were identified using Illumina deep sequencing data generated from leaves of droughted sweet orange plants grafted on the drought-tolerant rootstock ‘Rangpur’ lime. The reliability of our transcriptomic data was supported by detection of 41,827 (94.2%) unique transcripts out of 44,387 currently known sweet orange transcripts [27], and by the results of qPCR analysis (Table 1). They revealed a total of 1764 transcripts showing significant variation of expression in response to the drought stress conditions tested, which is far higher than in the previous studies [20, 21]. Our results show that the fraction of the sweet orange transcriptome regulated by drought stress is relatively small (4.2%), mostly represented by upregulated genes (61.3%) and functionally diverse (Figs. 1 and 2).

A striking leaf physiological trait of drought tolerance exhibited by sweet orange plants grafted on the drought-tolerant ‘Rangpur’ lime, but not by those grafted on the drought-sensitive ‘Flying Dragon’, is their increased cell wall elasticity (decreased ε) under drought [22], which contributes to the maintenance of cell turgor or symplast volume. Therefore, DEGs related to the cell wall metabolism are strong candidate genes for drought tolerance in citrus. In fact, 32 DEGs putatively involved in the cell wall metabolism were identified by RNA-Seq analysis (Table 2), with most of them showing significantly higher expression levels in the drought-tolerant (‘Rangpur’) than in the drought-sensitive (‘Flying Dragon’) rootstock (Table 3). These include DEGs coding for expansins (EXPs), PGs, PEIs, BXLs and PLLs, which are cell wall modifying proteins that modulate cell wall extensibility (EXPs, PEIs and BXLs) or plasticity/rheology (PGs and PLLs), contributing to the cell wall loosening [28]. They also include DEGs coding for AGPs and PRPs, which are structural cell wall proteins that have been proposed to act as plasticisers to loosen the pectin network or as stabilisers of the cell wall during times of cell stress or expansion [28, 29]. Involvement of these cell-wall related proteins in plant response and tolerance to drought stress has been reported in some previous studies. For instance, the *Arabidopsis* ortholog of *EXLB1* was shown to exhibit an early upregulation in to moderate drought stress, which was interpreted as an important strategy of drought acclimation by cell wall adjustment [30]. A pepper *PEI* gene (*CaPMEI1*) was shown to be induced by drought stress and its overexpression has conferred enhanced drought tolerance in transgenic *Arabidopsis*, as evidenced by their reduced transpiration and enhanced root elongation under drought conditions [31]. The mandarin ortholog of *BXL1* was shown to be highly induced by drought stress in roots [21]. Therefore, the differential expression of all these cell wall-related protein-encoding genes in rootstock varieties of contrasting drought tolerance makes them novel candidate genes for drought tolerance in citrus.

Some of the upregulated genes related to the metabolism of carbohydrates encode orthologs BFRUCT3, SPS1F, SUS3 and SUS4 (Additional file 4: Table S3), whose products of their enzymatic activities, glucose and sucrose [32–34], have been shown to increase significantly in leaves of drought-stressed sweet orange grafted on the drought-tolerant ‘Rangpur’ lime rootstock [35]. These sugars have been shown to play a pivotal role as osmoprotectants, helping to stabilise cellular membranes and to maintain cell turgor, a prerequisite for survival under stress conditions [36]. The downregulation of genes related to starch synthesis and degradation, such as those coding for HXK2, APS1, APL3, GBSS1 and BAM3 [37], is also in agreement with reductions in the starch content previously observed in leaves of sweet orange grafted on ‘Rangpur’ lime subjected to moderate water deficit [9], which occur probably as a consequence of the low photosynthesis.

Upregulation of genes coding for the antioxidant enzymes SOD and APX, and the carotenoid biosynthesis enzyme CHY1 (Additional file 4: Table S3) correlates with our previous observation that the drought tolerant ‘Rangpur’ roostock induced an efficient control of oxidative stress, which avoided damages to its photosynthetic apparatus and helped to maintain the cellular homeostasis under drought [22]. The mandarin ortholog of *CHY1* was shown to be induced by drought stress in leaves and roots and characterized as a novel molecular response of drought tolerance in citrus [21], since it is involved in the biosynthesis of zeaxanthin, a protective xanthophyll of the photosynthetic apparatus and a major precursor of abscisic acid (ABA).

Downregulation of all genes related to light reactions (Additional file 4: Table S3), such as *PSBA*, *PSBO*, *PSBP* and *CAB*, also corroborates with our previous findings that ‘Rangpur’ lime induced a decrease in the chlorophyll content in leaves of drought-stressed sweet orange, as indicated by the significant increase in the minimum fluorescence (F_0_) of chlorophyll *a* under drought conditions [22]. The loss of chlorophyll during periods of drought has been described as an important mechanism for photosynthetic photoprotection in plants, allowing less light absorption [38].

DEGs related to cellular response that were modulated by drought stress included homologues to well known genes involved in both biotic and abiotic stress response and redox regulation, as well as homologues to previously unknown genes associated with cell division, cell cycle and development (Fig. 2b and Additional file 5: Table S4). HSPs, ERDs, PMTs and miraculin are some of the well kwown gene products involved in the stress cellular responses in citrus and other plants [20, 21, 39, 40].

DEGs involved in the hormonal signaling pathways of ABA, auxin, ethylene, jasmonic acid (JA), salicylic acid (SA) and brassinosteroids were also identified (Fig. 2c and Additional file 6: Table S5). The ABA-regulated homologs of *HVA22*, *MARD1*, *AREB2* and *PP2C*s were highly induced by drought stress, whereas a downregulation was observed for *ABA DEFICIENT 1/2*, *CsPYL4* and *CsPYL5* (Additional file 6: Table S5). A positive regulation of *CsPP2CA* genes (e.g. *ABI1*, *ABI2*, *CsHAB1*) concomitantly with the downregulation of *CsPYR/PYL/RCAR* genes (e.g. *CsPYL4* and *CsPYL5*) in response to ABA accumulation during fruit ripening and leaf dehydration has been previously reported in sweet orange [41]. Taken together, our results suggest that these DEGs act as central elements in the signaling pathway and perception of ABA in citrus. On the other hand, except for a gene coding for the ethylene-responsive nuclear protein (ERT2), all the other genes related to the ethylene signaling pathway were downregulated by drought stress (Additional file 6: Table S5). These results suggest that drought inhibits the ethylene signaling in citrus, as previously reported for mandarin [21].

TFs have been shown to play important roles in drought stress response and tolerance via regulating downstream stress-responsive genes. Therefore, DEGs related to TFs also represent good candidate genes for drought tolerance in citrus. More than one hundred DEGs coding for TFs distributed in the major TF families (e.g. MYB, AP2/ERF, WRKY, bZIP, NAC, ZF, bHLH, HB, ARF and NF-Y) were identified in the present study (Fig. 2c; Additional file 7: Table S6), with most of them showing an upregulated expression in response to drought stress (Table 4). Two downregulated *MYB* DEGs found in the present study, *EARLY FLOWERING MYB* (*EFM*) and *MYB308-LIKE* (Additional file 7: Table S6), were previously identified in another study as sweet orange *CsMYB129* and *CsMYB64,* respectively, and shown to be downregulated in callus treated with mannitol [42]. *EFM* is an important flowering regulator that directly represses FLOWERING LOCUS T (FT) expression in the leaf vasculature [43]. Similarly, one upregulated *bZIP* DEG found in the present study, orange1.1g013223m (Additional file 7: Table S6), was previously characterized in *P. trifoliata* and its overexpression in tobacco increased drought tolerance of transgenic plants, possibly by inducing the synthesis of protective compounds, such as polyamines, or proteins, such as LEAs [44]. We also found an upregulated *AP2/ERF* DEG coding for DREB2A (Additional file 7: Table S6), which has been widely recognized as a key regulator in response to drought and temperature stresses, with high potential to increase plant tolerance to these stresses [45].

*BEE 3-LIKE*, *WRKY40* and *NAC2* were the three most upregulated TFs found in the present study (Table 1). Their differential expression between the rootstock varieties of contrasting drought tolerance (Table 3) has rendered them strong candidate genes for drought tolerance in citrus. Previous studies have shown that the *Arabidopsis* ortholog of *BEE 3-LIKE* (*BRASSINOSTEROID ENHANCED EXPRESSION 3-LIKE*) is a brassinosteroid (BR)-induced early response *bHLH* gene that also is antagonistically regulated by ABA, suggesting that it may function as a signaling intermediate in multiple pathways [46], whereas the hybrid poplar (*Populus alba* × *P. glandulosa*) ortholog of *BEE 3-LIKE* (*PagBEE3L*) was shown to induce plant growth and biomass production in transgenic poplar plants by increasing the xylem cell proliferation in stem tissue [47]. The *Arabidopsis* ortholog of *NAC2*, *ATAF1*, was shown to enhance the drought tolerance when overexpressed in transgenic *Arabidopsis* plants via stomatal regulation and induction of stress-responsive genes, such as *ADH1*, *RD29A* and *COR47* [48]. The Meiwa kumquat (*Fortunella crassifolia*) ortholog of *WRKY40* (*FcWRKY40*) was showed to be induced by salt, salicylic acid (SA), cold and ABA, but repressed by dehydration, and its overexpression in tobacco increased the tolerance of the transgenic plants to oxidative stress and the mRNA abundance of genes coding for peroxidase (POD) and catalase (CAT) [49]. The functional roles of the respective sweet orange orthologs of *BEE 3-LIKE*, *WRKY40* and *NAC2* remain to be elucidated.

Upstream of TFs, PKs regulate various signal transduction pathways in abiotic stress responses via perception of stress signals and activation of downstream signaling pathways by phosphorylating specific target proteins. Thus, they also represent good candidate genes for drought tolerance in citrus. We have found nearly one hundred DEGs coding for PKs, distributed in several families (Fig. 2d; Additional file 7: Table S6), with most of them also showing an upregulated expression in response to drought stress (Table 4). Two upregulated *PK*s belonging to the STK (orange1.1g019628m) and MAP kinase (orange1.1g023609m) families (Additional file 7: Table S6) are orthologs of the *Arabidopsis* SRK2C and MPK6, respectively. SRK2C is a SnRK2 protein involved in ABA signaling that significantly increase drought tolerance by upregulating many stress-responsive genes, such as *RD29A*, *COR15A*, and *DREB1A/CBF3* [50]. *MPK6* was shown to be induced in response to various abiotic and biotic stresses and it is considered a universal regulator in plant stress responses [51]. The differential expression of *WAKL8*, *WAKL2* and *WAK2* between rootstocks of contrasting drought tolerance (Table 3) also highlight these *WAK*s as relevant candidate genes for drought tolerance in citrus. WAK2 binding to native pectins on the cell wall is required for cell expansion during development, while its binding to pectin fragments like oligo-galacturonides (OGs) activates a stress response pathway [52].

## Conclusion

**Fig. 3:**
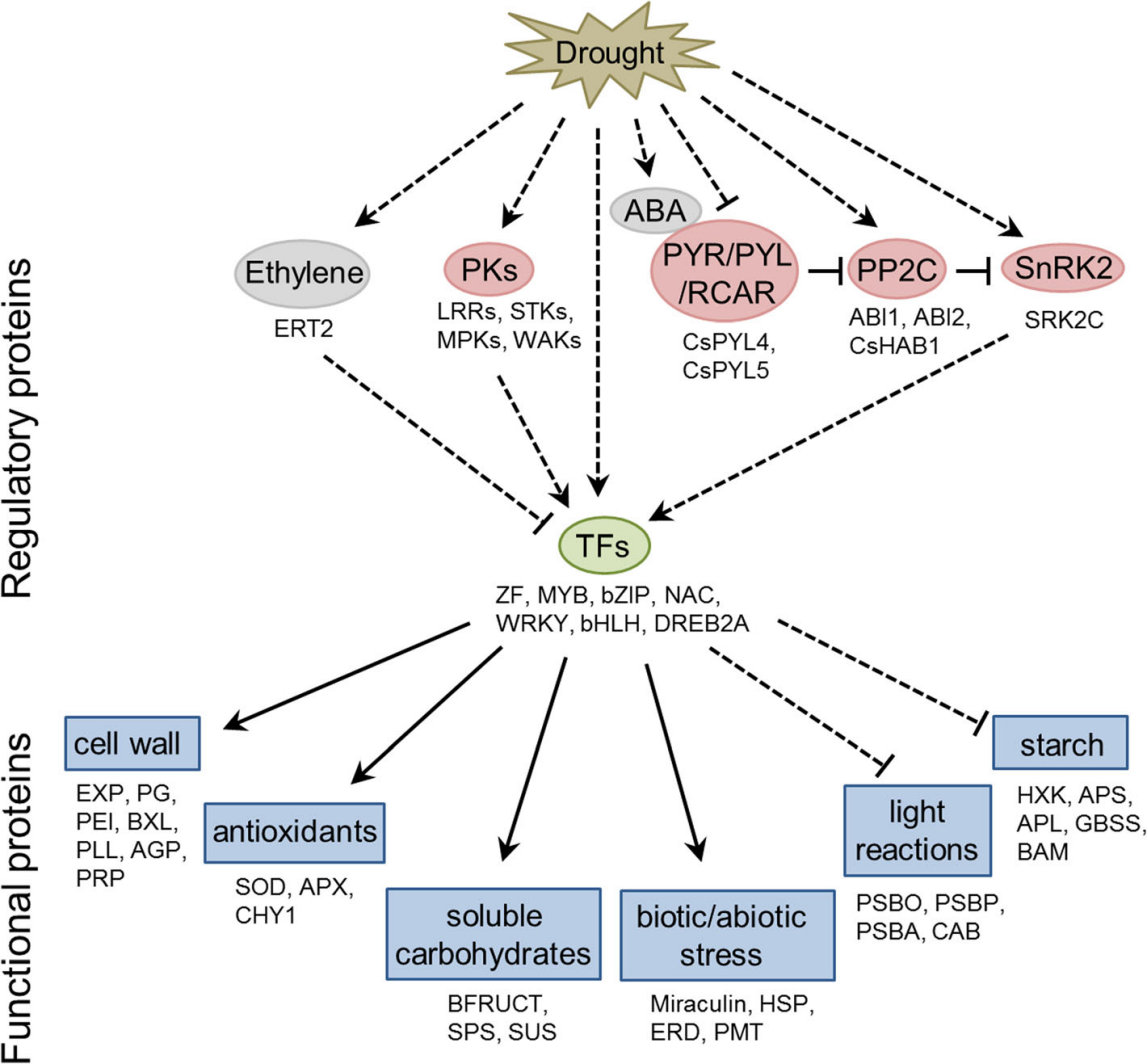
A schematic model of the rootstock-induced transcriptional response associated with drought tolerance in leaves of sweet orange. Ovals represent proteins or hormones and squares represent processes. Representative proteins are shown below the corresponding ovals and squares. Dotted lines represent indirect connections and solid lines represent direct connections. Abbreviations: ABA: abscisic acid; ABI1: ABA-insensitive 1; ABI2: ABA-insensitive 2; AGP: arabinogalactan protein; APL, ADP-glucose pyrophosphorylase large subunit; APS: ADP-glucose pyrophosphorylase; APX: ascorbate peroxidase; BAM: beta-amylase; BFRUCT: beta-fructosidase; bHLH: basic helix-loop-helix motif; BXL: β-xylosidase; bZIP: basic region/leucine zipper motif; CAB: chlorophyll a/b binding protein; CHY1: beta-carotene hydroxylase 1; CsHAB1: *Citrus sinensis* hypersensitive to ABA 1; CsPYL4: *Citrus sinensis* pyrabactin resistance 1-like 4; CsPYL5: *Citrus sinensis* pyrabactin resistance 1-like 5; DREB2A: dehydration-responsive element binding protein 2A; ERD: early responsive to dehydration; ERT2: ethylene-responsive nuclear protein 2; EXP: expansin; HSP: heat shock protein; GBSS: granule bound starch synthase; HXK: hexokinase; LRRs: leucine rich repeats; MPKs: mitogen-activated kinases; MYB: myeloblastosis; NAC: NAM/ATAF/CUC; PEI: pectinesterase inhibitor; PG: polygalacturonase; PKs: protein kinases; PLL: pectin lyase-like; PMT: putrescine N-methyltransferase; PP2C: protein phosphatase type 2C; PRP: proline-rich protein; PSBA: photosystem II reaction center protein D1; PSBO: photosystem II subunit O; PSBP: photosystem II subunit P; PYR/PYL/RCAR: pyrabactin resistance 1/PYR1-like/regulatory components of ABA receptor; SnRK2: SNF1- related protein kinase 2; SOD: superoxide dismutase; SPS: sucrose phosphate synthase; SRK2C: SNF1-related protein kinase 2C; STKs: Serine/Threonine-protein kinases; SUS: sucrose synthase; TFs: transcription factors; WAKs: wall-associated kinases; WRKY: tryptophan-arginine-lysine-tyrosine domain; ZF: zinc finger

The use of deep sequencing technology allowed to characterize the genome-wide molecular responses associated with the rootstock-induced drought tolerance in sweet orange. This analysis revealed that only a relatively small (4.2%) but functionally diverse fraction of the sweet orange transcriptome, with functions in metabolism, cellular responses and regulation, showed a significant variation of expression in response to the drought stress conditions tested. Taken together with the comparative expression analysis between sweet orange plants grafted on drought-tolerant and -sensitive rootstocks, as well as with the previously reported physiological and molecular responses of drought tolerance in citrus, the results suggest that drought tolerance in sweet orange involves the transcriptional activation of genes related to the metabolisms of cell wall, soluble carbohydrates and antioxidants and also of well known genes involved in biotic and abiotic stress responses, and the downregulation of genes involved in the starch metabolism, light reactions and ethylene signaling pathway. The regulatory pathways that modulate these downstream responses include several drought-regulated TFs, PKs and ABA signaling proteins. A schematic model depicting the transcriptional responses associated with the rootstock-induced drought tolerance in sweet orange is shown in Fig. 3. The present study provides a useful reference for further exploration of the functions of candidate genes and applications on the genetic improvement of citrus rootstocks.

## Methods

### Plant material

Leaf samples of twelve-month-old sweet orange [*Citrus sinensis* (L.) Osbeck var. ‘Westin’] grafted onto ‘Rangpur’ lime (*Citrus limonia* Osbeck, ‘Santa Cruz’ selection) or ‘Flying Dragon’ trifoliate orange [*Poncirus trifoliata* (L.) Raf.], subjected to control and drought stress treatments as previously reported [22], were used in the present study. These plants were obtained from the Citrus Germplasm Bank of Embrapa Cassava & Fruits (Cruz das Almas, BA, Brazil). The leaf predawn water potentials (Ψ_w_) in the control and drought-stress treatments were maintained at − 0,2–0,5 MPa and − 1,5 MPa, respectively. Five leaves (technical replicates) of three plants (biological replicates) were collected from each treatment and immediately frozen in liquid nitrogen.

### RNA isolation and RNA-Seq preparation

Total RNA was isolated from five leaves of each plant with the RNAqueous® kit (Applied Biosystems, Foster City, USA), following the manufacturer’s instructions. RNA was sequentially treated with DNase I (100 U g^− 1^ total RNA) (FPLC pure, Amersham Pharmacia Biotech, Piscataway, USA), according to the manufacturer’s instructions. The quality and integrity of the extracted RNA were evaluated by 1% (*w*/*v*) agarose gel analysis and the concentration measured on a NanoDrop 2000 spectrophotometer (NanoDrop Technologies, Wilmington, DE). One or two replicate RNA extractions were carried out in each plant for the construction of one (LC1) or two (LC3 and LC4) independent replicate cDNA libraries for control and drought treatments, respectively (Additional file 1: Table S1). Each cDNA library contained a pool of RNA in equal proportions from three plants per treatment. The RNA samples were sent to Macrogen Inc. (South Korea) for mRNA purification, cDNA library construction and sequencing using the platform Illumina.

### Sequence analysis

The 101 bp reads were collected and the sequences were mapped in the *Citrus sinensis* reference genome available in https://phytozome.jgi.doe.gov/pz/portal.html [53], using software TopHat. A quantitative assessment of the transcripts was used to calculate the levels of differential expression between control and drought treatments and significance levels using the Cuffdiff software, according to the procedures described by Coqueiro et al. [54]. The differentially expressed (*P* ≤ 0.001, fold-change ≥ + 2 and ≤ − 2) transcripts were recorded and automatically categorized using GO (Gene Ontology - http://www.blast2go.com/b2ghome). The functions of the identified genes were evaluated by comparing with the *A. thaliana* database (https://arabidopsis.org). MapMan software (https://mapman.gabipd.org) was also used as a tool for visualizing the functional classes significantly affected by drought treatment under diagrams of metabolic processes or other routes.

### Expression analysis by quantitative real-time PCR (qPCR)

Total RNA isolation and cDNA synthesis were performed as described previously [22]. Reverse transcription (RT) was performed using the RETROscript kit (Ambion, Austin, USA). Reactions were performed on a Stratagene Mx 3005P (Agilent Technologies) thermocycler containing the MxPro-Mx 3005P software, using the Maxima™ SYBR Green/ROX qPCR Master Mix kit (Thermo Scientific). Genes used as endogenous controls (reference genes) were glyceraldehyde-3-phosphate dehydrogenase C2 (GAPC2) and ubiquitin-protein ligase (UPL) [55]. Primer sequences are detailed in Additional file 8: Table S7. Reactions were carried out in triplicates, in a volume of 22 μL, containing 100 ng of cDNA, 1 μL of each specific primer, 10 μmol.L^− 1^ and 11 μL of Maxima® SYBR Green/ROX qPCR Master Mix (2X) (Fermentas, Maryland, USA). The amplification reactions were performed under the following conditions: (1) pretreatment at 50 °C for 2 min, (2) activation of Taq DNA polymerase at 95 °C for 10 min, (3) denaturation at 95 °C for 15 s, (4) annealing at 60 °C for 30 s, (5) extension at 60 °C for 1 min. The steps 3–5 were repeated for 30 cycles. To test the specificity of the primers, the products were analyzed by dissociation curve. For quantification of the expression, the 2^-ΔΔCt^ method [56] was used, considering the mean of the Ct values of the three replicates. Pearson correlations were calculated to compare the levels of gene expression measured by RNA-Seq and qPCR. Student’s *t* test was used to compare the gene expression levels between sweet orange plants grafted on ‘Rangpur’ lime and ‘Flying Dragon’ trifoliate.

## Additional files

Additional file 1: **Table S1.** Throughput and quality of RNA-Seq data. (DOCX 12 kb)

Additional file 2: **Table S2–1.** Differentially expressed genes that were upregulated in leaves of drought-stressed sweet orange (*Citrus sinensis*) grafted on ‘Rangpur’ lime. (XLSX 71 kb)

Additional file 3: **Table S2–2.** Differentially expressed genes that were downregulated in leaves of drought-stressed sweet orange (*Citrus sinensis*) grafted on ‘Rangpur’ lime. (XLSX 50 kb)

Additional file 4: **Table S3.** Differentially expressed genes related to metabolic pathways in leaves of drought-stressed sweet orange (*Citrus sinensis*) grafted on ‘Rangpur’ lime. (XLSX 19 kb)

Additional file 5: **Table S4.** Differentially expressed genes related to cellular response in leaves of sweet orange (*Citrus sinensis*) grafted on ‘Rangpur’ lime. (XLSX 13 kb)

Additional file 6: **Table S5.** Differentially expressed genes related to the hormonal signaling pathways in leaves of sweet orange (*Citrus sinensis*) grafted on ‘Rangpur’ lime. (XLSX 12 kb)

Additional file 7: **Table S6.** Differentially expressed genes related to transcription factors and protein kinases in leaves of sweet orange (*Citrus sinensis*) grafted on ‘Rangpur’ lime. (XLSX 23 kb)

Additional file 8: **Table S7.** Primer sequences used for validation in qPCR. (XLSX 12 kb)

## Abbreviations

ABA: Abscisic acid
ABF: ABRE-binding factor
ABI1: ABA-insensitive 1
ABI2: ABA-insensitive 2
ABRE: ABA-responsive element
ADH1: Alcohol dehydrogenase 1
AGP1: Arabinogalactan protein 1
AP2/ERF: APETALA2/ethylene responsive factor
APL: ADP-glucose pyrophosphorylase large subunit
APS: ADP-glucose pyrophosphorylase
APX: Ascorbate peroxidase
AREB2: ABRE-binding protein 2;
BAM3: Beta-amylase 3
BEE 3-LIKE: Brassinosteroid enhanced expression 3-Like
BFRUCT3: Beta-fructosidase 3
bHLH: Basic helix-loop-helix motif
BR: Brassinosteroid
BXL1: β-xylosidase 1
bZIP: Basic region/leucine zipper motif
CAB: Chlorophyll a/b binding protein
CAT: Catalase
CESA: Cellulose synthase
CHY1: Beta-carotene hydroxylase 1
COR15A: Cold-related 15A
COR47: Cold-related 47
CTR1: Constitutive triple response 1
DEGs: Differentially expressed genes
DREB1A/CBF3: Dehydration-responsive element binding protein 1A/C-repeat (CRT) binding factor 3
DREB2A: Dehydration-responsive element binding protein 2A
ERD: Early responsive to dehydration
ERT2: Ethylene-responsive nuclear protein 2
EST: Expressed sequence tag
EXLB1: Expansin-like B1
EXP: Expansin
FT: Flowering locus T
GA: Gibberellic acid
GAP2C: Glyceraldehyde-3-phosphate dehydrogenase C2
GAP3C: Glyceraldehyde-3-phosphate dehydrogenase C3
GBSS1: Granule bound starch synthase 1
GGT: Glycogenin glucosyltransferase
GO: Gene ontology
HAB1: Hypersensitive to ABA 1
HSP: Heat shock protein
HVA22: *Hordeum vulgare* abscisic acid-induced protein 22
HXK2: Hexokinase 2
IAA: Indole-3-acetic acid
JA: Jasmonic acid
LEA: Late embryogenesis abundant
LOX2: Lipoxygenase 2
LRR: Leucine rich repeat
MAP: Mitogen-activated protein
MARD1: Mediator of ABA-regulated dormancy 1
MEKK1: Mitogen-activated kinase kinase
MPK: Mitogen-activated kinase
MPK6: MAP kinase 6
MYB: Myeloblastosis
NAC: NAM/ATAF/CUC
PEG: Polyethylene glycol
PEI 54-like: Pectinesterase inhibitor 54-like
PEI 6-like: Pectinesterase inhibitor 6-like
PEP2Cs: Protein phosphatases type 2C
PEPCK: Phosphoenol pyruvate carboxykinase
PG: Polygalacturonase
PKs: Protein kinases
PLL1: Pectin lyase-1 like
PMT: Putrescine N-methyltransferase
POD: Peroxidase
PRP4: Proline-rich protein 4
PSBA: Photosystem II reaction center protein D1
PSBO: Photosystem II subunit O
PSBP: Photosystem II subunit P
PYL: PYR1-like
PYR: Pyrabactin resistance 1
qPCR: Quantitative real-time PCR
RCAR: Regulatory components of ABA receptor
RCI2: Rare-cold-inducible 2
RD29A: Responsive to dehydration 29A
ROS: Reactive oxygen species
RPS13A: Small subunit ribosomal protein S13A
RWC^TLP^: Relative water content at turgor loss point
SnRK2: SNF1-Related protein kinase 2
SOD: Superoxide dismutase
SPS1F: Sucrose phosphate synthase 1F
SRK2C: SNF1-Related protein kinase 2C
STK: Serine/Threonine-protein kinase
SUS: Sucrose synthase
T6P: Trehalose-6-phosphate
TCA: Tricarboxylic acid
TFs: Transcription factors
TIR1: Transport inhibitor response 1
TPS: Trehalose-6-phosphate synthase
UPL: Ubiquitin-protein ligase
WAK: Wall-associated kinase
WAKL: WAK-like
WRKY: Tryptophan-arginine-lysine-tyrosine domain
WUE: Water use efficiency
ZF: Zinc finger
