# Supplementary material for: Rootstock-induced molecular responses associated with drought tolerance in sweet orange as revealed by RNA-Seq
Source: BMC Genomics. 2019 Feb 6;20:110. doi: 10.1186/s12864-019-5481-z (PMC6364419; doi:10.1186/s12864-019-5481-z)
Supplement: Supplementary file 1 — Table S1. Throughput and quality of RNA-Seq data. (DOCX 12 kb) [file 12864_2019_5481_MOESM1_ESM.docx]

| Sample | Total Bases | Read Count | N (%) | GC (%) | Q20 (%) | Q30 (%) |
| --- | --- | --- | --- | --- | --- | --- |
| LC1 (irrigated) | 5,984,788,734 | 59,255,334 | 0.005 | 46.51 | 96.41 | 91.55 |
| LC3 (drought-stressed) | 6,745,911,604 | 66,791,204 | 0.005 | 43.74 | 96.64 | 91.59 |
| LC4 (drought-stressed) | 6,042,892,014 | 59,830,614 | 0.005 | 43.79 | 96.61 | 91.53 |

**Table S1.** Throughput and quality of RNA-Seq data
